# Effective isolation of Sertoli cells from New Zealand rabbit testis

**DOI:** 10.5455/javar.2021.h505

**Published:** 2021-06-18

**Authors:** Wen-Qian Zhu, De-Cai Yang, Yu Jiang, Ning-Ning Cai, Rui Yang, Xue-Ming Zhang

**Affiliations:** Animal Histology and Embryology, College of Veterinary Medicine, Jilin University, Changchun, Jilin Province, China

**Keywords:** Enrichment, identification, rabbit, separation, Sertoli cells

## Abstract

**Objective:**

Sertoli cells (SCs) are important sustentacular cells in the seminiferous tubules of the testis. Isolation and identification of SCs are the premise for studying their functions. Since New Zealand rabbit is a stable strain which is widely used for biomedical research and animal farming, this study aimed to develop a simple and effective protocol for SC isolation in New Zealand rabbits.

**Materials and Methods:**

The SCs of three 30-day-old New Zealand rabbits were isolated by incubation with enzymatic digestion I (Dulbecco’s modified Eagle medium supplemented with 1 mg/ml collagenase IV and 50 μg/ml DNase I) and digestion II (digestion I + 1 mg/ml hyaluronidase + 1 mg/ml trypsin), as well as differential plating. The cells were enriched and identified by using immunocytochemical staining and reverse transcription polymerase chain reaction analysis.

**Results:**

Homogeneous cells were obtained. They presented the typical large cell body and an irregular pyramidal shape after differential plating and passaging. These cells expressed mRNA of the SC marker sex-determining region Y-box 9 (*SOX9*) instead of the Leydig cell marker *StAR*. Immunocytochemically, they are positive of *SOX9*, GATA binding protein 4, and androgen-binding protein.

**Conclusion:**

The SCs were enriched from the testicular tissues of prepubertal New Zealand rabbits by a simple and effective protocol, which provides a basis for further theoretical researches and practical applications.

## Introduction

Sertoli cells (SCs) are a type of multifunctional cells that interact directly with spermatogenic cells in the seminiferous epithelium and play a pivotal part in maintaining the spermatogenic microenvironment and coordinating spermatogenesis. The number of SCs and their structure, as well as function, determines the population number of spermatogenic cells and other somatic cells during testicular development, therefore affecting the spermatogenic efficiency and reproductive ability of male gonads [1].

The SCs create the main components of the blood–testis barrier (BTB), the tight junction, and anchoring junction with testicular cells, so that they can provide physical support and a stable milieu for germ cells [2] and protect meiosis and haploid germ cells from the host’s autoimmune system [3]. Furthermore, SCs also deliver a variety of cytokines to regulate spermatogenesis in an endocrine and paracrine manner. For example, the anti-Mullerian hormone (AMH) secreted by SCs is closely related to the proliferation and differentiation of testicular germ cells [4,5]. The AMH is susceptible to the co-modulation of transcription factors of sex-determining region Y-box 9 (*SOX9*) and GATA binding protein 4 (GATA4). The androgen levels in the testis relate to the androgen-binding protein (ABP), which is also produced by SCs; in turn, androgen regulates the spermatogenesis and sperm maturation [6,7]. The SCs were also discovered having the ability of immunomodulation that can protect co-transplanted cells exerting normal properties after transplantation [8]. Hence, an *in vitro* method for effectively isolating and enriching SCs will greatly promote their use in multiple fields. Previously, we successfully obtained the seminiferous epithelial suspension from the cryopreserved bovine testicular tissues through a two-step enzymatic procedure [9,10] and further isolated and enriched bovine SCs [11,12]. Since New Zealand rabbits are being increasingly used as experimental animals for research on topics ranging from human reproduction studies to preclinical pharmacological studies [13,14], we aimed to develop a simple and effective protocol for SC isolation from this animal in the present study.

## Materials and Methods

### Animals and reagents

Three healthy male (30-day-old) New Zealand rabbits were acquired from Charles River Laboratory (Beijing, China). Animal usage was approved by the Jilin University Institutional Animal Care and Use Committee (SY201906007). All rabbits were kept in individual cages at room temperature (RT) under a 12 h/12 h light/dark cycle and fed a normal diet and water. The rabbits were euthanized by an overdose of anesthetics (105 mg/kg ketamine + 15 mg/kg xylazine). To make sure the rabbits are healthy before euthanasia, their feeding and drinking behaviors were monitored daily. The chemicals were bought from Sigma-Aldrich (St. Louis, MO) and the basic medium was purchased from Gibco (CA), unless otherwise stated. Anti-*SOX9* was bought from Abcam (Cambridge, UK), anti-GATA4 was bought from Affinity Biosciences (OH), and anti-ABP was provided by Bioss (Beijing, China). 

### Isolation and culture of SCs

SC isolation by a two-step enzymatic procedure was carried out as reported [9]. Briefly, the testes were excised and the epididymides and tunica albuginea were removed under sterile conditions using fine scissors and tweezers. Phosphate buffered saline (PBS) containing 5% penicillin–streptomycin combination was used to clean the testes. Next, tissues were mechanically and vigorously dissociated into discrete seminiferous tubules in tubes containing PBS. The tissues were sedimented for 15 min. Then the precipitated tubular fragments were collected after decanting the supernate. The tubular segments were transferred into a 10 ml tube containing enzymatic digestion I [Dulbecco’s modified Eagle medium (DMEM) supplemented with 1 mg/ml collagenase IV (Biosharp, Anhui, China) + 50 μg/ml DNase I] and incubated at 37°C for 30 min in a water bath. Subsequently, the samples were washed, centrifuged to deposit, and transferred into another 10 ml tube containing enzymatic digestion II (digestion I supplemented with 1 mg/ml trypsin and 1 mg/ml hyaluronidase). The contents were digested for 15 min under the similar conditions and 10% (v/v, similarly hereinafter) fetal bovine serum (FBS; Biological Industries, Israel) was used to block enzyme activity. The digested tissues were filtrated using a 40-μm nylon mesh. After removing the undigested clumps, the single-cell suspension was cultured in six-well plates. The suspended cells were removed after 5–6 h cultivation in 5% CO_2_ at 37°C and the adherent cells were cultured in DMEM supplemented with 10% FBS, 1% non-essential amino acid, 1% GlutaMAXTM supplement, and 1% penicillin–streptomycin. The differential plating was proceeded as described [14] and the medium was changed every 2 days.

### Immunocytochemistry

The third passage (P3) of the above enriched cells was seeded in 96-well plates, cultivated for 48 h, and fixed in PBS-buffered 4% paraformaldehyde for 30 min at RT. Next, they were permeabilized for 20 min using 0.1% Triton X-100, incubated for 30 min in 3% H_2_O_2_, and 60 min with 5% bovine serum albumin (Biosharp, Anhui, China). The samples were treated for 8 h at 4°C with primary antibodies (*SOX9*, GATA4, and ABP; all 1:200) before incubation for 30 min at RT with biotinylated goat anti-rabbit IgG and another 30 min incubation with SABC reagent (Boster, Wuhan, China). The samples were washed thrice with PBS. Diaminobenzidine tertrahydrochloride (DAB Horseradish Peroxidase Color Development Kit, Boster) was used as chromogen. The samples were observed and imaged under a light microscope.

### Reverse transcriptase polymerase chain reaction (RT-PCR)

The P3 cells (1×10^6^) were digested with 0.25% Trypsin-EDTA (Gibco). Total RNA was extracted by using TRIzon Reagent (CoWin Biosciences, Jiangsu, China) and quantified by Thermo Nanodrop 2000 (Gene Company Limited, Shanghai, China). The cDNA synthesis was carried out using TransScript^®^ All-in-One First-Strand cDNA Synthesis SuperMix for PCR (TransGen, Beijing, China). The primers (Table 1) were designed for *GAPDH*, *SOX9*, and *StAR*. The SC marker *SOX9* and the Leydig cell marker *StAR* were detected by RT-PCR, with *GAPDH* as the control. The reactions were conducted in a 20 μl system which contains 10 μl SYBR green master mix (TaKaRa, Beijing, China), 1 μl cDNA, 7 μl RNase-free water, 1 μl forward primer (10 μM), and 1 μl reverse primer (10 μM) for each gene. The PCR conditions are as follows: initialization at 94°C for 5 min, followed by 35 PCR cycles of denaturation at 94°C for 30 sec, anneal at 60°C for 30 sec, extension at 72°C for 30 sec, and final extension at 72°C for 10 min, then cooling at 4°C. The PCR products were revealed by electrophoresis on a 1% (w/v) agarose gel.

**Table 1.** Primers for RT-PCR analysis.

Gene	Primer sequence (5’→3’)	Product sizes (bp)
*GAPDH*	F-GTCGGAGTGAACGGATTTGG	394
	R-GGTTCACGCCCATCACAAAC	
*SOX9*	F-ATGACCGACGAGCAGGAGAAGG	239
	R-ACCAGCGTCCAGTCGTAGCC	
*StAR*	F-GAGGGCTGGAAGGAGGAGAACC	228
	R-CTCGTGCGTGATGACCGTGTC	

## Results

### Enrichment and morphology of rabbit SCs

**Figure 1.**
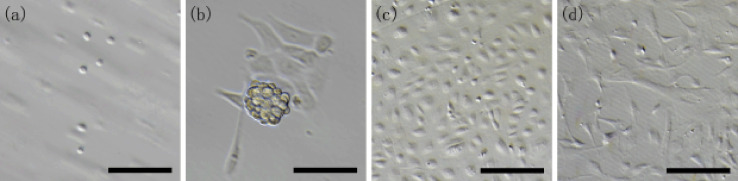
Enrichment and culture of rabbit SCs. (a) Rabbit seminiferous epithelial cells; (b) grape cluster-like germline colonies and spreading somatic cells after being cultured for 20 h; (c) enriched SCs cultured for 3 days; and (d) P3 SCs. Scale bars: 50 μm.

The seminiferous epithelial single-cell suspension from prepubertal rabbits mainly consists of numerous testicular somatic cells and germ cells. These cells were round with varied sizes, highly refractive, and uniformly suspended in the media (Fig. 1a). After being cultured for 5–6 h, some germ cells formed grape-like cluster colonies sporadically attached on the adherent somatic cells, which appeared in a polygonal shape with multiple cytoplasmic processes (Fig. 1b). Hence, differential plating and serial passaging were used to eliminate the cells remaining in suspension and those slightly attached. After being cultured for 2–3 days, the primary cells showed a relative regular oval shape (Fig. 1c). Following expansion to P3, the adherent cells presented as an irregular pyramidal shape (Fig. 1d).

### Characterization of the enriched SCs

**Figure 2.**
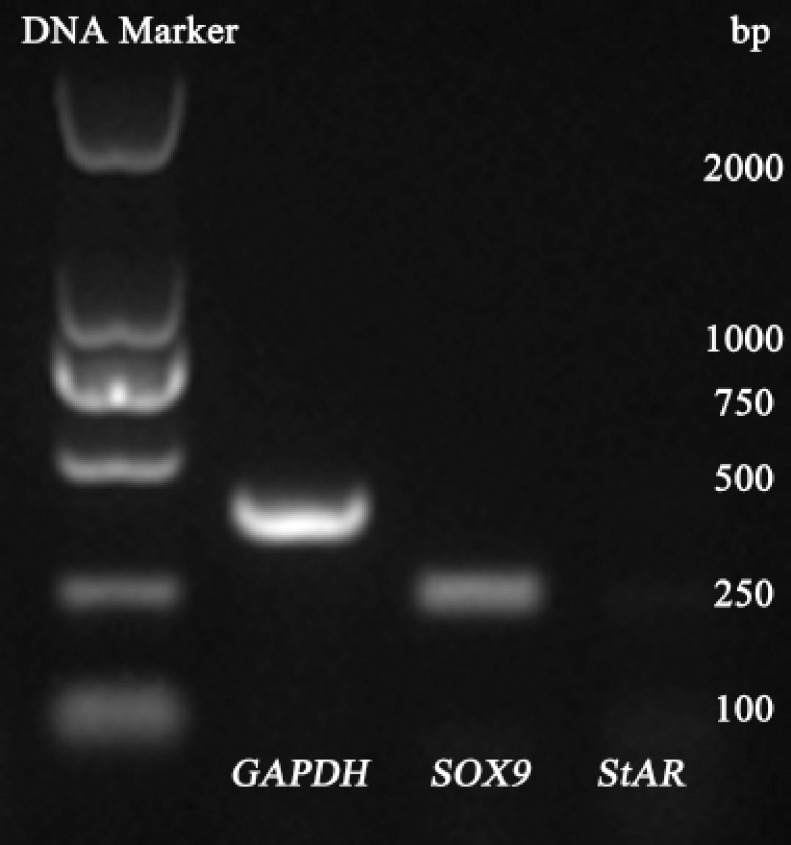
Genotype of cultured P3 SCs with *GAPDH* as control.

**Figure 3.**
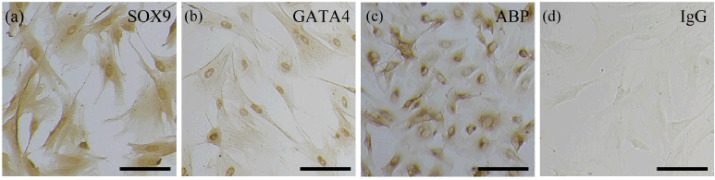
Immunocytochemical staining. (a) *SOX9*, (b) GATA*4*, and (c) ABP staining with (d) isotype control. Scale bars: 100 μm.

RT-PCR analysis showed that there was mRNA transcript expression of the SC marker *SOX9* instead of the Leydig cell marker *StAR* in the enriched cells (Fig. 2). We further carried out immunocytochemical staining using specific antibodies to verify the protein expression of the SC marker *SOX9*, GATA4, and ABP in these cells. Specifically, the enriched cells were all positive for these markers (Fig. 3). Their cellular localizations were in the nuclear and cytoplasm for *SOX9* (Fig. 3a), strong and exclusively in the nuclear for GATA4 (Fig. 3b), and in the cytoplasm and cell membrane for ABP (Fig. 3c), compared with the isotype control (Fig. 3d).

## Discussion

The New Zealand rabbit is a stable mammalian inbreeding species. It is widely used in experimental biomedical studies due to its advantages, such as small size, long life span, and easy feeding [8,13,16]. Therefore, exploration of the reproduction mechanism using this animal is of great importance. In the testis, SCs are the most important somatic cells which form the main structure of the BTB, provide a stable and immunoprivileged microenvironment, and secret modulating factors for normal spermatogenesis. Currently, SC study is also one of the research hotspots in cell engineering and tissue engineering techniques, as well as regenerative medicine, especially in the field of clinical transplantation [8]. Adult SCs are post-mitotic terminally differentiated cells with a stable number but no proliferative capacity [17]. The seminiferous cords of neonatal animal testis mainly consist of immature SCs and germ cells [9], which make it possible to obtain SCs with a higher proliferative ability theoretically. However, the neonatal rabbit testis is very small and attaches on the peritoneum, making it difficult to obtain enough tissue. Thus, the testis of 30-day-old rabbits was used in the present study.

**Figure 4.**
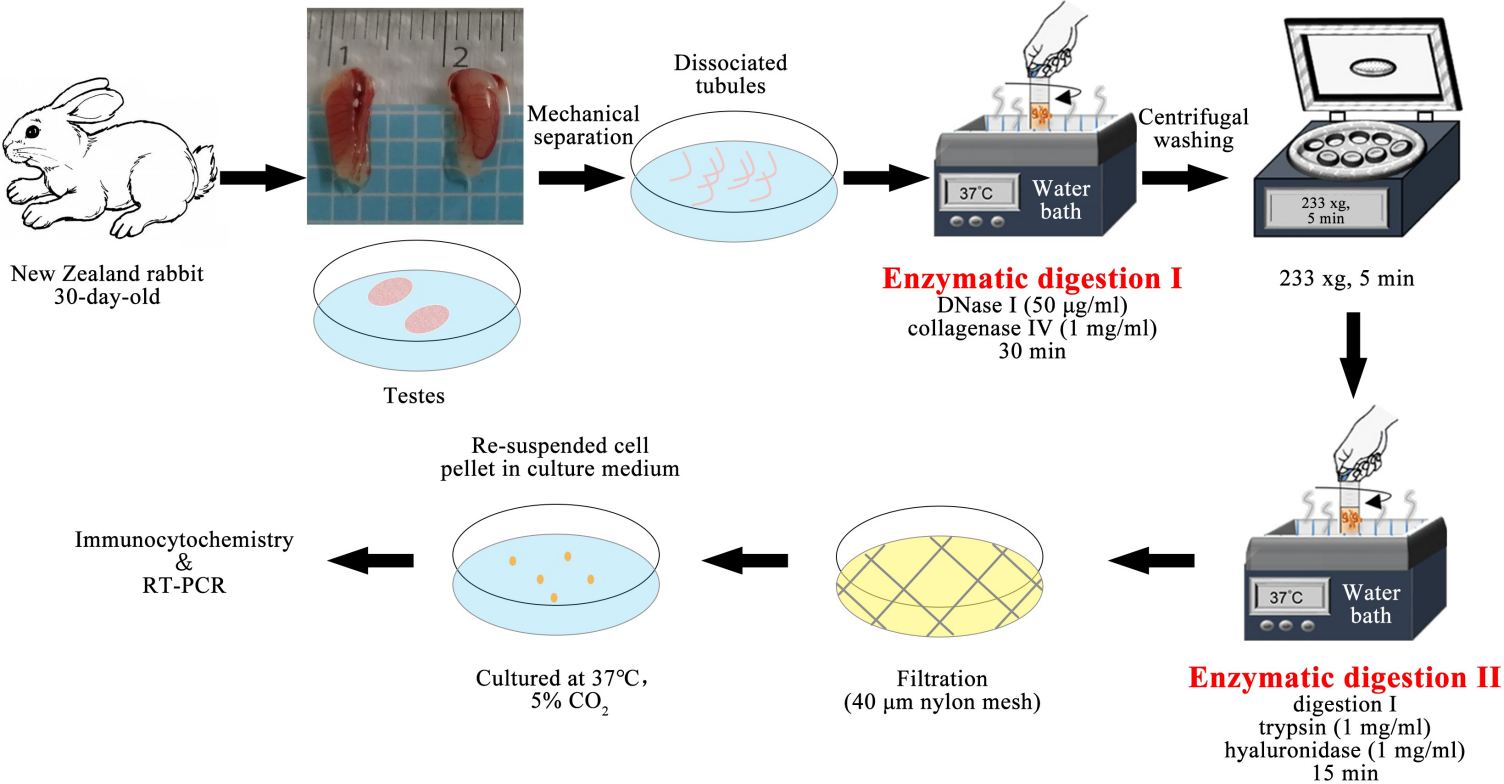
Protocol of SC isolation and identification.

The isolation technique used in this study is basically similar with that described previously for the enrichment of bovine SCs [11,12]. The mechanical separation and sedimentation eliminated the most interstitial tissues and obtained purified seminiferous tubules. Two-step enzyme digestion was used to effectively dissociate the seminiferous epithelium and reduce the enzymatic damage to cells. Impurities could be further eluted by low-speed centrifugation and deposition. The filtration using a nylon mesh removed the undigested clumps and guaranteed single-cell suspension. Finally, the differential plating and serial passaging eliminated germ cells and gave rise to a very high purity of SCs, because germ cells have a delayed adherent property and cannot survive for a long time in common medium. The enriched SCs could robustly proliferate and reach 80% confluence within 2–3 days during cultivation. 

To further confirm the identity of these cells, RT-PCR analysis and immunocytochemistry were carried out. Our previous study indicated that germ cells cannot be maintained *in vitro *for a long time under common conditions [10]; therefore, the marker gene and protein in germ cells are not investigated here. Transcriptionally, the SC marker gene *SOX9* instead of the Leydig cell marker *StAR* was detected, suggesting that the enriched cells were SCs and maintained their original characteristics in culture with the elimination of Leydig cells. Previously, Grieco et al. [18] and Banco et al. [19] investigated the histological and immunohistochemical characteristics of rabbit SCs. Here we demonstrate that these cells also expressed the SC marker protein *SOX9*, GATA4, and ABP, which further confirm their identity and extend our knowledge about their molecular biological features. The present study is helpful to promote spermatogenesis mechanism studies using these cells as an *in vitro* model, and also benefits the related cell engineering, tissue engineering techniques, as well as regenerative medicine studies. Nevertheless, their biological functions, such as structural supports, factor secretion, and immunoprotection for the germ cell development, still need to be further revealed in our next step.

## Conclusion

Altogether, we isolated and enriched SCs from the testicular tissues of prepubertal New Zealand rabbits by a simple and effective protocol (Figure 4). These cells presented typical morphological features and molecular markers. The present study provides a solid basis for related theoretical research and practical application in biology of reproduction.

## List of abbreviations

ABP, androgen -binding protein; AMH, anti-Mullerian hormone; BTB, blood–testis barrier; DMEM, Dulbecco’’s modified Eagle medium; GATA4, GATA binding protein 4; h, hour; min, minute; P3, third passage; PBS, phosphate buffered saline; RT, room temperature; RT-PCR, reverse transcription- polymerase chain reaction; sec, second, SCs, Sertoli cells; *SOX9*, sex -determining region Y-box 9; EDTA, Ethylene diamine tetraacetic acid.
